# Dental and skeletal changes in patients with mandibular retrognathism following treatment with Herbst and pre-adjusted fixed appliance

**DOI:** 10.1590/2176-9451.19.1.046-054.oar

**Published:** 2014

**Authors:** Fabio de Abreu Vigorito, Gladys Cristina Dominguez, Luís Antônio de Arruda Aidar

**Affiliations:** 1 PhD in Orthodontics, School of Dentistry, University of São Paulo (USP).; 2 Full professor, Department of Orthodontics, University of São Paulo (USP).; 3 Full professor, Department of Orthodontics, University of Santa Cecília (UNISANTA).

**Keywords:** Angle Class II malocclusion, Orthopedics, Orthodontics

## Abstract

**Objective:**

To assess the dentoskeletal changes observed in treatment of Class II, division 1 malocclusion patients with mandibular retrognathism. Treatment was performed with the Herbst orthopedic appliance during 13 months (phase I) and pre-adjusted orthodontic fixed appliance (phase II).

**Methods:**

Lateral cephalograms of 17 adolescents were taken in phase I onset (T_1_) and completion (T_2_); in the first thirteen months of phase II (T_3_) and in phase II completion (T_4_). Differences among the cephalometric variables were statistically analyzed (Bonferroni variance and multiple comparisons).

**Results:**

From T_1_ to T_4_, 42% of overall maxillary growth was observed between T_1_ and T_2_ (P < 0.01), 40.3% between T_2_ and T_3_ (P < 0.05) and 17.7% between T_3_ and T_4_ (n.s.). As for overall mandibular movement, 48.2% was observed between T_1_ and T_2_ (P < 0.001) and 51.8% between T_2_ and T_4_ (P < 0.01) of which 15.1% was observed between T_2_ and T_3_ (n.s.) and 36.7% between T_3_ and T_4_ (P < 0.01). Class II molar relationship and overjet were properly corrected. The occlusal plane which rotated clockwise between T_1_ and T_2_, returned to its initial position between T_2_ and T_3_ remaining stable until T_4_. The mandibular plane inclination did not change at any time during treatment.

**Conclusion:**

Mandibular growth was significantly greater in comparison to maxillary, allowing sagittal maxillomandibular adjustment. The dentoalveolar changes (upper molar) that overcorrected the malocclusion in phase I, partially recurred in phase II, but did not hinder correction of the malocclusion. Facial type was preserved.

## Abstract (pt)

**Objetivo:**

avaliar as alterações dentoesqueléticas observadas no tratamento da má oclusão de Classe II com retrognatismo mandibular, realizado com aparelho ortopédico de Herbst durante 13 meses (Fase I) e aparelho ortodôntico fixo pré-ajustado (Fase II).

**Métodos:**

foram obtidas telerradiografias laterais de 17 adolescentes, ao início (T_1_), final da Fase I (T_2_), primeiros 13 meses da Fase II (T_3_) e término da Fase II (T_4_). As diferenças entre as variáveis cefalométricas foram analisadas estatisticamente (variância e comparações múltiplas de Bonferroni).

**Resultados:**

de T_1_ a T_4_, do total da projeção da maxila, 42% foram observados de T_1_ a T_2_ (p < 0,01); 40,3% de T_2_ a T_3_ (p < 0,05); e 17,7% de T_3_ a T_4 _(n.s.). Do total da projeção da mandíbula, foi notado 48,2% de T_1_ a T_2_ (p < 0,001) e 51,8% de T_2_ a T_4_ (p < 0,01), sendo 15,1% (n.s.) de T_2_ a T_3_, e 36,7% de T_3_ a T_4_ (p < 0,01). A relação molar e a sobressaliência foram corrigidas idealmente. Em T_4_, todos apresentavam características de oclusão normal. O plano oclusal que de T_1_ a T_2_ rotacionou no sentido horário, de T_2_ a T_3_ retornou aos valores iniciais, mantendo-se estável até T_4_. A inclinação do plano mandibular, responsável pela caracterização do tipo facial, não alterou em nenhum tempo.

**Conclusão:**

a mandíbula cresceu significativamente mais que a maxila, favorecendo o ajuste sagital maxilomandibular. As mudanças dentárias (molares superiores), que sobrecorrigiram a má oclusão na Fase I, recidivaram parcialmente na Fase II, sem comprometer a correção da má oclusão. O tipo facial foi preservado.

## INTRODUCTION

Growing patients with Class II malocclusion and mandibular retrognathism may be treated with a variety of techniques, as described in the literature. Some of the techniques include treatment performed with an orthopedic phase employing appliances such as the Herbst. This treatment has been widely studied by Pancherz^1-4^ and other researchers^5-22^ who took several aspects into consideration and revealed that this type of treatment not only represents an alternative to the correction of Class II malocclusion, but also preserves the stomatognathic system. However, with regard to Brazilian individuals, these results are questioned: Are treatment effects skeletal or dentoalveolar? Is the mandibular growth curve modified when stimulated by the Herbst appliance? Are the obtained results lost after the appliance is removed? The complexity of clarifying the referred doubts lays in the difficulty of performing longitudinal studies in homogeneous casuistries. With a view to eliminating the tendency towards including only successful cases and, thus, confuse the results, the ideal would be that prospective studies were conducted with groups of consecutive patients. From this point of view, in 2007, a study^23^ was carried out to assess and compare, in patients treated during growth spurt, the dentoskeletal changes observed in the Herbst active phase and during a period of same duration after the appliance had been removed. The obtained results were the motivation to perform the present study which aims at assessing full treatment performed in adolescents in two phases: phase I - orthopedic with Herbst appliance and phase II - orthodontic with pre-adjusted fixed appliance.

## MATERIAL AND METHODS

The sample comprised 17 Brazilian adolescent patients (12 men and 5 women), with mean age of 12 years and 4 months ± 1 year and 2 months, and bone age corresponding to the growth spurt, as revealed by a hand-wrist radiograph. The patients were selected according to the following inclusion criteria: individuals with mandibular retrognathism and Angle Class II, division 1 malocclusion greater than half-cusp (> 3 mm); individuals with overjet > 5 mm (permanent dentition); with model discrepancy under 4 mm; with clinical recommendation for mandibular advancement to be performed with functional orthopedic appliance. Individuals with absence of teeth, dental fractures and dental caries were excluded. Treatment was carried out in two phases. Initially, the orthopedic phase (phase I) performed with Herbst functional orthopedic appliance placed onto acrylic splints associated with maxillary expansion screw.^24^ The objective was to correct the transversal discrepancy,^25^ activating the expansion screw during the first month of treatment. The appliance was made according to a wax bite registration obtained with 6 mm of initial advancement, and progressive advancements of 2 mm every 2 months, according to individual needs. This phase lasted for an average of 13.9 ± 2.1 months. Thereafter, the orthodontic phase (phase II) was performed with pre-adjusted fixed appliance and aimed at leveling and aligning the upper and lower teeth as well as at obtaining functional occlusion with adequate overjet and overbite. This phase lasted for 46 months.

**Figure 1:**
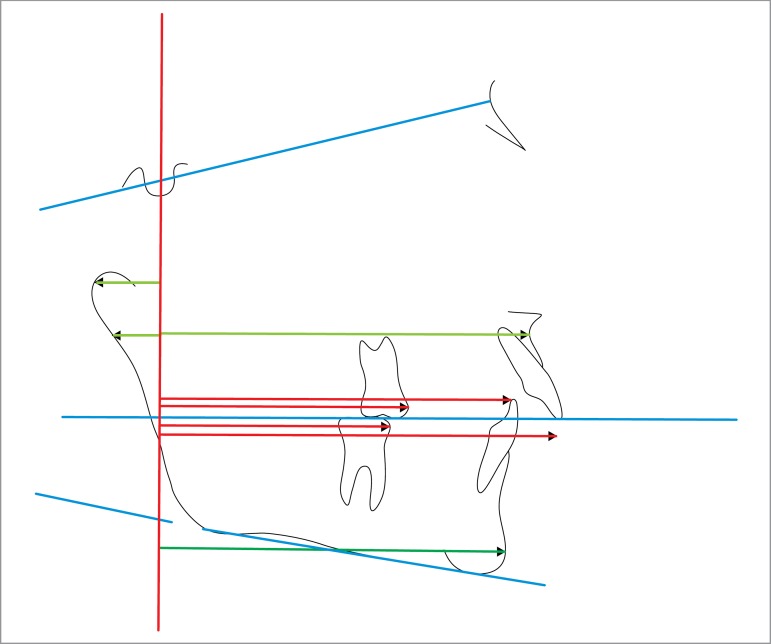
Analysis of sagittal changes (SO-analysis) of Pancherz.

**Figure 2:**
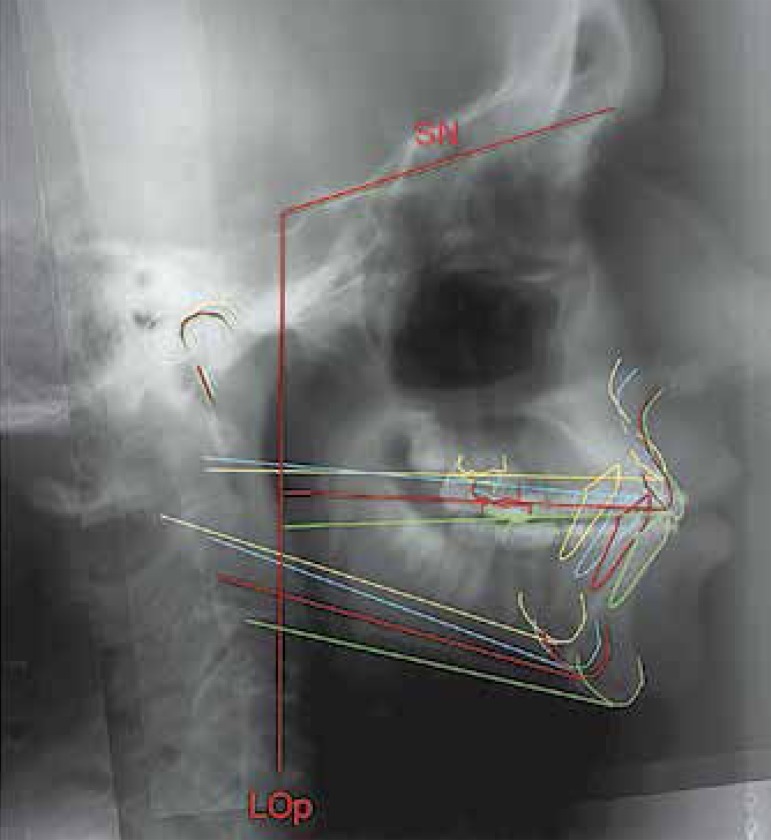
Superimposition of tracings (according to analysis of Pancherz(4)), of one of the patients from the sample, in all four observation stages: T1 = yellow, T2 = blue, T3 = red and T4 = green.

Complete orthodontic documentation (panoramic and hand-wrist radiographs, lateral and frontal cephalograms; intra and extraoral photographs; study casts) was prepared for all patients at four stages: T_1_, immediately before treatment onset; T_2_, after 13 months using the Herbst appliance, which represented the end of phase I; T_3_, 13 months after phase II or orthodontic phase had begun; and T_4_, phase II completion, totalizing a period of 33 months. All 68 lateral cephalograms were manually traced by the same operator at monthly intervals. They were analyzed with regard to the cephalometric variables of sagittal changes analysis (SO-analysis) suggested by Pancherz^4^ (Figs 1 and 2).

Patients' guardians signed an informed consent form, agreeing with all stages of the study and the posterior disclosure of results. The project was approved by the Institutional Review Board of the School of Dentistry/USP and registered under protocol 109/06.

## STATISTICAL ANALYSIS

Method error assessment (Dahlberg^26^) was performed in 11.8% of the sample.

The values of each measure and the relation of each moment assessed by means and standard deviations were expressed and compared to the measurements taken between the moments of assessment using the analysis of variance carried out with repeated measures. For measurements that presented statistically significant differences between the moments of assessment, Bonferroni multiple comparisons were performed. They revealed in which moments these differences occurred. The tests were performed with a significance level set at 5%.

## RESULTS

For a better understanding of the characteristics of each moment of growth (Fig 2) and the differences between them, the results are presented in three tables. Table 1 presents the measures, the relation between measurements at each moment of assessment and the result of the analysis of variance.

**Table 1:** Measures and relations between values obtained at each moment of assessment and result of the analysis of variance.

Variable	Orthopedic phase		Orthodontic phase		N	p
	T_1_	T_2_	T_3_	T_4_		
	Mean ± S.D.	Mean ± S.D.	Mean ± S.D.	Mean ± S.D.		
SN.PM	32.59 ± 5.42	32.56 ± 5.59	31.88 ± 5.36	32.03 ± 6.26	17	0.439
ui/Lop	90.77 ± 5.18	90.47 ± 6.55	92.15 ± 6.78	93.79 ± 6.28	17	<0.001
li/Lop	81.62 ± 5.95	87.82 ± 6.50	87.97 ± 6.60	90.06 ± 6.20	17	<0.001
um/Lop	57.12 ± 4.31	57.21 ± 5.02	59.56 ± 4.91	62.35 ± 5.45	17	<0.001
lm/Lop	55.53 ± 4.70	61.32 ± 5.24	62.59 ± 5.14	65.59 ± 5.61	17	<0.001
ss/Lop	81.24 ± 3.47	82.50 ± 3.94	83.71 ± 4.06	84.24 ± 4.40	17	<0.001
pg/Lop	83.15 ± 5.30	87.59 ± 5.85	88.59 ± 6.04	91.68 ± 6.19	17	<0.001
ar/Lop	10.59 ± 3.73	10.24 ± 3.70	10.44 ± 4.12	9.88 ± 4.14	17	0.241
co/Lop	12.94 ± 3.62	12.91 ± 3.58	13.29 ± 3.63	13.56 ± 4.08	17	0.319
SN.LO	20.29 ± 3.72	23.15 ± 4.63	20.79 ± 4.47	19.06 ± 4.72	17	<0.001
ui/Lop-li/Lop	9.15 ± 2.74	2.65 ± 1.23	4.18 ± 1.20	3.74 ± 0.90	17	<0.001
um/Lop-lm/Lop	1.59 ± 1.61	-4.12 ± 2.10	-3.03 ± 1.58	-3.24 ± 1.15	17	<0.001
pg/Lop+ar/Lop	93.74 ± 5.18	97.82 ± 5.76	99.03 ± 6.74	101.56 ± 7.66	17	<0.001
pg/Lop+co/Lop	96.09 ± 5.12	100.50 ± 5.69	101.88 ± 6.62	105.24 ± 7.95	17	<0.001
ui/Lop-ss/Lop	9.53 ± 2.70	7.97 ± 3.12	8.44 ± 3.28	9.56 ± 2.93	17	0.022
li/Lop-pg/Lop	-1.53 ± 5.83	0.24 ± 5.86	-0.62 ± 5.61	-1.62 ± 5.63	17	0.031
um/Lop-ss/Lop	-24.12 ± 2.24	-25.29 ± 2.30	-24.15 ± 2.18	-21.88 ± 2.71	17	<0.001
lm/Lop-pg/Lop	-27.62 ± 3.74	-26.26 ± 4.20	-26.00 ± 4.46	-26.09 ± 4.92	17	0.008

The results of Bonferroni multiple comparisons are presented in Table 2, whereas the results presenting differences in the relation between measures are shown in Table 3.

**Table 2:** Result of Bonferroni multiple comparisons for measurements that presented differences during treatment.

Variable	Comparison	Mean difference	Standard error	p	CI (95%)	
					Lower	Upper
ui/Lop	T_ 1_ - T_2_	0.29	0.56	> 0.999	-1.40	1.99
	T_ 1_ - T_3_	-1.38	0.76	0.516	-3.66	0.89
	T_ 1_ - T_4_	-3.03	0.70	0.003	-5.14	-0.92
	T_2_ - T_3_	-1.68	0.49	0.020	-3.14	-0.21
	T_2_ - T_4_	-3.32	0.73	0.002	-5.51	-1.14
	T_3_ - T_4_	-1.65	0.52	0.034	-3.20	-0.10
li/Lop	T_ 1_ - T_2_	-6.21	0.48	< 0.001	-7.66	-4.75
	T_ 1_ - T_3_	-6.35	0.65	< 0.001	-8.30	-4.41
	T_ 1_ - T_4_	-8.44	0.86	< 0.001	-11.01	-5.87
	T_2_ - T_3_	-0.15	0.56	> 0.999	-1.83	1.54
	T_2_ - T_4_	-2.24	0.79	0.072	-4.61	0.14
	T_3_ - T_4_	-2.09	0.59	0.016	-3.86	-0.31
um/Lop	T_ 1_ - T_2_	-0.09	0.40	> 0.999	-1.29	1.11
	T_ 1_ - T_3_	-2.44	0.44	< 0.001	-3.75	-1.13
	T_ 1_ - T_4_	-5.24	0.74	< 0.001	-7.45	-3.02
	T_2_ - T_3_	-2.35	0.37	< 0.001	-3.46	-1.25
	T_2_ - T_4_	-5.15	0.85	< 0.001	-7.70	-2.59
	T_3_ - T_4_	-2.79	0.65	0.003	-4.74	-0.85
lm/Lop	T_ 1_ - T_2_	-5.79	0.50	< 0.001	-7.29	-4.30
	T_ 1_ - T_3_	-7.06	0.57	< 0.001	-8.76	-5.36
	T_ 1_ - T_4_	-10.06	1.02	< 0.001	-13.13	-6.99
	T_2_ - T_3_	-1.27	0.45	0.073	-2.61	0.08
	T_2_ - T_4_	-4.27	0.98	0.003	-7.22	-1.31
	T_3_ - T_4_	-3.00	0.69	0.003	-5.07	-0.93
ss/Lop	T_ 1_ - T_2_	-1.27	0.30	0.004	-2.17	-0.36
	T_ 1_ - T_3_	-2.47	0.45	< 0.001	-3.82	-1.12
	T_ 1_ - T_4_	-3.00	0.66	0.002	-4.99	-1.01
	T_2_ - T_3_	-1.21	0.37	0.032	-2.33	-0.08
	T_2_ - T_4_	-1.74	0.64	0.094	-3.67	0.20
	T_3_ - T_4_	-0.53	0.43	> 0.999	-1.81	0.75
pg/Lop	T_ 1_ - T_2_	-4.44	0.53	< 0.001	-6.02	-2.86
	T_ 1_ - T_3_	-5.44	0.71	< 0.001	-7.57	-3.31
	T_ 1_ - T_4_	-8.53	1.11	< 0.001	-11.86	-5.20
	T_2_ - T_3_	-1.00	0.46	0.280	-2.40	0.40
	T_2_ - T_4_	-4.09	1.00	0.005	-7.10	-1.08
	T_3_ - T_4_	-3.09	0.70	0.003	-5.19	-0.99
SN.LO	T_ 1_ - T_2_	-2.85	0.85	0.023	-5.40	-0.31
	T_ 1_ - T_3_	-0.50	0.75	> 0.999	-2.76	1.76
	T_ 1_ - T_4_	1.24	0.78	0.801	-1.12	3.59
	T_2_ - T_3_	2.35	0.32	< 0.001	1.39	3.32
	T_2_ - T_4_	4.09	0.86	0.001	1.51	6.67
	T_3_ - T_4_	1.74	0.61	0.068	-0.09	3.56

**Table 3:** Result of Bonferroni multiple comparisons for relations between measures that presented differences during treatment.

Variable	Comparison	Mean difference	Standard error	p	CI (95%)	
					Lower	Upper
ui/Lop-li/Lop	T_1_ - T_2_	6.50	0.71	< 0.001	4.37	8.64
	T_1_ - T_3_	4.97	0.78	< 0.001	2.64	7.31
	T_1_ - T_4_	5.41	0.75	< 0.001	3.16	7.66
	T_2_ - T_3_	-1.53	0.49	0.041	-3.01	-0.05
	T_2_ - T_4_	-1.09	0.43	0.129	-2.37	0.20
	T_3_ - T_4_	0.44	0.30	0.963	-0.46	1.34
um/Lop-lm/Lop	T_1_ - T_2_	5.71	0.47	< 0.001	4.30	7.11
	T_1_ - T_3_	4.62	0.43	< 0.001	3.33	5.90
	T_1_ - T_4_	4.82	0.40	< 0.001	3.61	6.03
	T_2_ - T_3_	-1.09	0.24	0.002	-1.81	-0.37
	T_2_ - T_4_	-0.88	0.47	0.482	-2.30	0.54
	T_3_ - T_4_	0.21	0.33	> 0.999	-0.78	1.19
pg/Lop+ar/Lop	T_1_ - T_2_	-4.09	0.44	< 0.001	-5.42	-2.75
	T_1_ - T_3_	-5.29	0.69	< 0.001	-7.36	-3.23
	T_1_ - T_4_	-7.82	1.05	< 0.001	-10.98	-4.67
	T_2_ - T_3_	-1.21	0.56	0.286	-2.90	0.49
	T_2_ - T_4_	-3.74	0.96	0.008	-6.61	-0.86
	T_3_ - T_4_	-2.53	0.56	0.002	-4.22	-0.84
pg/Lop+co/Lop	T_1_ - T_2_	-4.41	0.37	< 0.001	-5.52	-3.30
	T_1_ - T_3_	-5.79	0.61	< 0.001	-7.63	-3.96
	T_1_ - T_4_	-9.15	1.12	< 0.001	-12.52	-5.77
	T_2_ - T_3_	-1.38	0.51	0.096	-2.93	0.16
	T_2_ - T_4_	-4.74	1.06	0.002	-7.91	-1.56
	T_3_ - T_4_	-3.35	0.71	0.001	-5.50	-1.21
ui/Lop-ss/Lop	T_1_ - T_2_	1.56	0.49	0.035	0.08	3.03
	T_1_ - T_3_	1.09	0.71	0.857	-1.04	3.21
	T_1_ - T_4_	-0.03	0.70	> 0.999	-2.12	2.06
	T_2_ - T_3_	-0.47	0.44	> 0.999	-1.80	0.86
	T_2_ - T_4_	-1.59	0.59	0.095	-3.36	0.18
	T_3_ - T_4_	-1.12	0.46	0.155	-2.49	0.25
li/Lop-pg/Lop	T_1_ - T_2_	-1.77	0.44	0.006	-3.08	-0.45
	T_1_ - T_3_	-0.91	0.76	> 0.999	-3.20	1.37
	T_1_ - T_4_	0.09	0.77	> 0.999	-2.24	2.41
	T_2_ - T_3_	0.85	0.59	> 0.999	-0.92	2.63
	T_2_ - T_4_	1.85	0.66	0.076	-0.13	3.84
	T_3_ - T_4_	1.00	0.37	0.092	-0.11	2.11
um/Lop-ss/Lop	T_1_ - T_2_	1.18	0.30	0.008	0.27	2.09
	T_1_ - T_3_	0.03	0.39	> 0.999	-1.13	1.19
	T_1_ - T_4_	-2.24	0.45	0.001	-3.60	-0.88
	T_2_ - T_3_	-1.15	0.33	0.019	-2.14	-0.15
	T_2_ - T_4_	-3.41	0.58	< 0.001	-5.16	-1.67
	T_3_ - T_4_	-2.27	0.58	0.008	-4.02	-0.51
lm/Lop-pg/Lop	T_1_ - T_2_	-1.35	0.41	0.026	-2.58	-0.12
	T_1_ - T_3_	-1.62	0.53	0.045	-3.21	-0.03
	T_1_ - T_4_	-1.53	0.62	0.156	-3.41	0.35
	T_2_ - T_3_	-0.27	0.26	> 0.999	-1.05	0.52
	T_2_ - T_4_	-0.18	0.47	> 0.999	-1.59	1.24
	T_3_ - T_4_	0.09	0.35	> 0.999	-0.97	1.15

## DISCUSSION

All patients that comprised this study presented, in T_1_, typical characteristics of Class II division 1 malocclusion, as confirmed by the initial cephalometric variables that describe the molar relationship (um/Lop - lm/Lop: 1.59 ± 1.61 mm) and the overjet (ui/Lop - li/Lop: 9.15 ± 2.74 mm). According to the inclusion criteria, all patients clinically presented mandibular retrognathism and accepted treatment that included mandibular advancement.

The results yielded by the present study are in agreement with previous studies that used similar methods.^9,12,23,27^ Both the maxilla (SS/Lop) and mandible (PPg/Lop) were anteriorly projected, but since mandibular growth increment was 3.5 times greater, there was a favorable sagittal maxillomandibular adjustment. In order to identify the contribution of mandibular growth, measurements of the absolute mandibular length (pg/Lop+co/Lop and pg/Lop+ar/Lop) were assessed and significant growth increment was observed, although the condylar (co/Lop) and articular (ar/Lop) points did not present any alterations.

The registered amount of skeletal growth allowed better a understanding of how the teeth varied in their sagittal spatial position. Overcorrection of the observed molar relationship (um/Lop-lm/Lop: 5.71 mm) was due to the association between maintenance of upper molars position (um/Lop: -0.09) while the maxilla was anteriorly projected (SS/Lop: -1.27 mm), and mesialization of lower molars (lm/Lop: -5.79 mm) along with mandibular anterior projection (pg/Lop: -4.44 mm). Overjet was significantly reduced from 9.5 mm to 2.65 mm, as a result of mandibular anterior projection (pg/Lop: -4.44 mm) and buccal inclination of lower incisors in their bone base (li/Lop: -6.21 mm). The mechanical effect observed in the inclination of lower incisors restricts the recommendation of this type of therapy to individuals who do not present increased inclination at treatment onset.

The occlusal plane (SN.LO), which in the beginning presented a mean value that is typical of a mesofacial pattern (32.59 ± 5.42º), was rotated clockwise (2.85º) by the presence of interocclusal acrylic splints. This might have caused the effect of molar intrusion, since, when the appliance was removed, an important posterior disocclusion was observed in all patients. This speculation can be done because, differently from the occlusal plane, the inclination of the mandibular plane (SN.PM) did not undergo any alterations, thus confirming that it was just a dentoalveolar effect and not a skeletal one, therefore, the facial type did not change.

In the following 13 months after the Herbst appliance had been removed, which corresponded to orthodontic treatment onset (T_2_-T_3_), the maxilla continued to be anteriorly projected (ss/Lop: -1.21 mm), whereas mandibular projection was little significant (pg/Lop: -1 mm). It was observed that partial recurrence of molar relationship (um/Lop - lm/Lop: -1.09 mm) occurred as a result of mesialization of upper molars (um/Lop-ss/Lop: -1.15 mm) along with non-significant mesialization of lower molars (lm/Lop-pg/Lop: -027). However, considering that a relation of overcorrection of molar relationship was observed in T_2_ (um/Lop - lm/Lop: -4.12 mm), this recurrence was favorable to adjust the molars in Class I relation (um/Lop - lm/Lop: -3.03 mm). Additionally, there was a partial recurrence of 1.53 mm in overjet (ui/Lop-li/Lop) as a result of differential growth of the maxilla, which led the upper incisors to occupy a more anterior spatial position (is/Lop: -1.68 mm). This could not have been due to the insignificant uprighting of lower incisors (li/Lop: -0.15 mm) because, in this case, they did not change their position (li/Lop-pg/Lop: 0.85 mm).

The occlusal plane (SL.LO) rotated counterclockwise, since, from T_2_ to T_3_, with the removal of the Herbst appliance, the molars were free from the interocclusal splints and, additionally, were actively leveled to the orthodontic appliance, restoring the vertical spatial position that they presented at treatment onset. These data corroborate data found in the literature,^9,10^ thus confirming that this movement happened without affecting the inclination of the mandibular plane (SN-PM), therefore, with preservation of facial type.

The complementary assessment carried out in this study, between the thirteen-month interval after removal of the Herbst appliance and the end of the active orthodontic treatment (T_3_-T_4_), showed that, while the maxilla was not significantly anteriorly projected (SS/Lop: -0.53 mm), the mandible resumed its growth (pg/Lop: -3.09 mm), significantly anteriorly projecting itself. Molar relationship (um/Lop-lm/Lop: 0.21 mm) remained stable in Class I. Moreover, no expressive changes were observed for the overjet (ui/Lop-li/Lop: 0.44mm).

Nevertheless, when analyzing the maintenance of dental stability, during a period in which there was significant expression of mandibular growth and absence of significant maxillary growth, it could be observed that tooth movement was compensatory, maintaining both molar and overjet relations. While the upper incisors (ui/Lop: -1.65 mm) and the upper molars (um/Lop: -2.79 mm) were anteriorly projected in the absence of significant maxillary growth (ss/Lop: -053 mm), the lower incisors (li/Lop: -2.09 mm) and lower molars (lm/Lop:-3 mm) were also spatially anteriorly projected, however, in association with significant mandibular growth (pg/Lop: -3.09 mm). Thus, it can be concluded by means of the differential calculus (dental movement minus skeletal movement) that only the upper molars had a significant movement of mesialization, regardless of the growth of its bone base (um/Lop-ss/Lop: -2.27 mm). This movement was necessary to maintain Class I molar relationship. The occlusal plane remained stable (SN.LO: 1.74º). This fact can be explained because in T_3_, the molars already presented interocclusal contact and there were no additional vertical movements until T_4_. The mandibular plane remained unchanged, revealing a uniform behavior during the entire treatment, thus, preserving facial type.

When considering the series of changes observed from the beginning to the end of treatment (T_1_-T_4_), it is verified that out of the total of maxillary anterior projection (3 mm), 42% happened during the orthopedic phase (T_1_-T_2_) and 58% during the orthodontic phase (T_2_-T_4_), of which the most part (40.3%) happened during the first 13 months (T_2_-T_3_) and the rest (17.7%), an insignificant increase, between T_3_ and T_4_. As shown in Tables 1 to 3, the mandibular anterior displacements (Pg/Lop) were compatible to the corresponding increment of the mandibular absolute growth (Pg/Lop + ar/Lop and Pg/Lop + co/Lop). When analyzing the variable Pg/Lop + co/Lop, it is verified that 48.2% of mandibular growth happened during the 13 months of the orthopedic phase (T_1_-T_2_) as a response to the stimulus provided by the Herbst appliance, in a period when the potential growth was intense; whereas 51.8% happened during the orthodontic phase (T_2_-T_4_). However, it must be emphasized that during the 13 months after the Herbst appliance was removed (T_2_-T_3_), there was growth deceleration, with slight, non-significant growth increment (15.1%) and, therefore, without anterior projection. Significant growth was soon resumed, expressing the remaining 36.7% in the following months until T_4_. This type of response agrees with previous studies.^4,9^ It was very important to assess the amount of growth during the orthodontic phase (T_2_-T_4_) as proposed in this study. Moreover, dividing observation into two periods, T_2_-T_3_ (13 months) and T_3_-T_4_ (33 months), was important to understand whether or not the curve of mandibular growth could modify its usual course before the stimulus given by the use of the Herbst appliance. Franchi et al^28^ claim that mandibular growth follows the physical growth spurt and it is characterized by a gradual increase in the amount of increments until it reaches its maximum, when the greatest amount of growth is expressed. Afterwards, it gradually decelerates again, however, linearly, until growth is complete. In the present study, it was observed that during the 13 months of stimulus (T_1_-T_2_) provided by the Herbst appliance, the increments were intense. Nevertheless, a deceleration in the following 13 months (T_2_-T_3_), and then, a resumption of growth (T_3_-T_4_), explain that the growth verified between T_1_ and T_2_ represents the favorable expression of the present growth potential, for being in its maximum (as revealed by the hand-wrist radiograph in T_1_), which is summed up to the anticipation of growth in the subsequent 13 months, which, without the use of the appliance, would not have manifested at that moment, thus, modifying the behavior of the descendant curve of growth spurt in adolescence.

As for growth complexity and mandibular spatial projection in the face, our results can be explained by those observed by Pancherz et al^29^ who assessed the "effective condylar growth" and its influence over the spatial position of the symphysis in the face. Their findings reveal that condylar growth triplicated during the active phase of six months in which the Herbst appliance was used, decelerated in a similar period after the removal of the appliance, and soon resumed its normal growth in the subsequent 30 months. Comparison between total mandibular and maxillary projection, from T_1_ to T_4_, revealed that the mandible (pg/Lop: 8.47 mm) was projected 2.8 times more than the maxilla (ss/Lop: 3 mm), a fact that favored sagittal maxillomandibular adjustment.

With regard to dentoalveolar correction of Class II malocclusion, a favorable response was observed between T_1_ and T_4_, i.e., Class II molar relationship and the increase in overjet that patients presented at treatment onset were ideally corrected. In T_4_, all of them showed characteristics of normal occlusion, with good molar relationship and adequate overjet, thus, achieving the purpose of the treatment. In order to produce such results, treatment evolved from sagittal overcorrection of molar relationship, which was associated with great reduction in overjet during the 13-month orthopedic phase; partially relapsed at the beginning of the orthodontic phase and became stable in the following 33 months until the end of the treatment.

Based on the aforementioned observations, it is important to emphasize that: First, the recurrence of the overcorrected molar relationship between T_2_ and T_3_ was necessary for molars to obtain cusp-to-fossa relationship instead of cusp-to-cusp, which probably contributed to offer the stability observed in the subsequent period. Additionally, despite being significant, the degree of overjet relapse registered between T_2_ and T_3_, did not prevent the values from being within the clinical parameters of normality by the end of the treatment. The second aspect is with regard to the stability observed in T_3_ and T_4_, a period of 33 months. The advantage of lasting nearly two times longer than each previous period allowed the stability of results to be assessed.

Clockwise rotation of the occlusal plane was significant during the orthopedic phase (T_1_-T_2_) and it happened as a result of the presence of interocclusal splints. In the subsequent phase (T_2_-T_3_), it rotated counterclockwise, therefore, relapsing by the removal of the splints and active orthodontic leveling, thus, restoring intermaxillary occlusal contacts. This pattern of counterclockwise rotation continued in the following 33 months, however, insignificantly. As for the changes that occurred in opposite directions, the comparison between orthopedic and orthodontic phases reveal that they did not present any adverse clinical effect, since the changes occurred without influencing the inclination of the mandibular plane. On the other hand, the occlusal plane restored its initial inclination in T_3_ and remained stable until T_4_. The mandibular plane (SN.PM), which defines the facial type, was maintained in all periods of assessment, a fact that is favorable to the stability achieved in the long term, all of which agreed with other authors in the literature.^9,30^

The size of the sample is a limitation of this study. However, it is of great value considering that it is a prospective study carried out with consecutive patients and that had never been performed with Brazilian patients. The results obtained from assessing these patients by means of the treatment protocol allowed us to visualize not only that the therapy applied was efficient, but also that the series of skeletal and dental changes observed did not cause a temporary impact, but an impact that is compatible with the conditions of stability in the long term. However, further studies are necessary to longitudinally assess the post-treatment phase. Finally, it is important to emphasize the undesirable effect that the use of the Herbst appliance can cause to individuals with increased buccal inclination of the lower incisors at treatment onset.

## CONCLUSIONS

Based on the results of treatment of adolescents with Class II malocclusion and mandibular retrognathism performed in two phases (Herbst and pre-adjusted orthodontic appliance) it is reasonable to conclude that both skeletal and dental changes, when performed together, allowed the correction of the malocclusion. The mandible grew significantly more than the maxilla, which favored sagittal maxillomandibular adjustment. The dental changes (distalization of upper molars) that overcorrected the malocclusion in phase I partially relapsed in phase II, without compromising the correction of the malocclusion. Th e facial type was preserved.
